# Development and application of a spray tip that enables electrocoagulation of a variety of tissues

**DOI:** 10.1016/j.heliyon.2023.e17771

**Published:** 2023-07-25

**Authors:** Satomi Iwai, Shou Kobayashi, Shinji Torai, Eiji Kobayashi

**Affiliations:** a Kitasato University School of Veterinary Medicine, Endowed Chair for the Promotion of Minipig Research, 35-1, Higashi 23, Towada City, Aomori, 034-8628, Japan; b Kobayashi Regenerative Research Institute, LLC, 1 Chayanochou, Wakayama-shi, Wakayama-ken, 640-8263, Japan; c Department of Kidney Regenerative Medicine, Industry-Academia Collaborative Department, The Jikei University School of Medicine, 3-25-8 Nishi-Shimbashi, Minato-ku, Tokyo, 105-8461, Japan

**Keywords:** Cauterization, Electrosurgical device, Hemostasis, New tip development, Seven-claw tip, Spray coagulation

## Abstract

**Background:**

Spray hemostasis is possible using a high-frequency power source from the tip of an electric scalpel; however, the difficulties regarding the uniformity and rapidity of the hemostasis surface remain. This study reports the development of a novel electrocoagulation device tip that can be used in endoscopic and robotic surgeries and can quickly coagulate and hemostat and easily adjust the extent of cauterization and hemostasis while minimizing the depth of thermal injury.

**Methods:**

The safety and efficacy of the hemostatic device were verified in a porcine model. A liver surface transection was conducted in vivo and the rapidity of the hemostatic effect of the device was observed. An extracted stomach, kidney, and liver were cauterized ex vivo by three operators with different surgical skills and the effects were analyzed pathologically. In addition, a sacrificed pig cadaver was used to achieve hemostasis at a renal transection site using the multi-spray endoscope tip.

**Results:**

An increase in the number of tip terminals expanded the cauterization surface and shortened the cauterization time. In parenchymatous organs, uniform cauterization was possible without increasing the depth of thermal injury. The cauterization depth did not depend on the operator's skill, and the spray coagulation was safe. The variable spray tip allowed for simple hemostasis during open and laparoscopic surgeries.

**Conclusions:**

This novel electrocoagulation device tip can be developed as a forceps that can change the spray range and can be used during laparoscopic and robotic surgeries.

## Highlights

• The seven-claw spray tip reduced the cauterization depth and tissue invasion compared to the one-claw spray tip.
• The seven-claw spray tip enabled stable cauterization techniques regardless of operator experience.
• The seven-claw spray tip can shorten hemostasis time by uniform cauterization over a wide area.
• It was experimentally demonstrated that simple and safe hemostasis was possible with a multiple-tip spray.

## 1 Introduction

Since their introduction in 1920 [1], electrosurgical devices have been used in over 80% of surgical procedures for incisions, hemostasis control, and cauterization [2]. An electric scalpel applies a high-frequency current to tissues, and the load or contact resistance generates a Joule heating effect that instantaneously heats the cells. This heat results in desiccation, vaporization, or charring of the target tissue yielding an incision effect. The intracellular water is evaporated, leading to the coagulation of proteins. The electrical discharge coagulation method, in which hemostasis is achieved directly with an electric scalpel, can stop bleeding in small blood vessels with a diameter ≤0.5 mm. The contact coagulation method, in which the blood vessel is clamped with hemostatic forceps to arrest the bleeding and then cauterized with an electric scalpel, is capable of stopping bleeding in blood vessels with a diameter ≤2 mm. Monopolar devices require a metal return electrode to be attached to the patient, and the operator can use the electric power from the high-frequency-generating power supply to easily make incisions and achieve coagulation with the tip of an electric scalpel. This reduces the blood loss during surgery. Furthermore, the coagulation mode of the electric scalpel can effectively coagulate surfaces that tend to bleed, such as venous sinuses, without contact using spray electric power [3]. A method of strengthening this non-contact coagulation spray with argon gas has been proposed and is widely used to achieve hemostasis using a flexible endoscope and during laparotomy [4]. However, due to the use of argon gas, this process is expensive [5]. The spray coagulation is conducted without the electric scalpel tip contacting the cauterized surface; therefore, the time required for coagulation and hemostasis may be problematic. In addition, the cauterization depth varies depending on the skill of the operator.

In this study, the effectiveness of a newly developed, multi-directional electrocoagulation spray tip was verified using a porcine parenchymal organ hemostasis model. In addition, the multi-directional electrocoagulation spray tip is foldable, allowing for its use during laparotomy as well as in endoscopic surgery.

## 2 Materials and methods

### 2.1 Creation of multi-tip spray tip

**Fig. 1:**
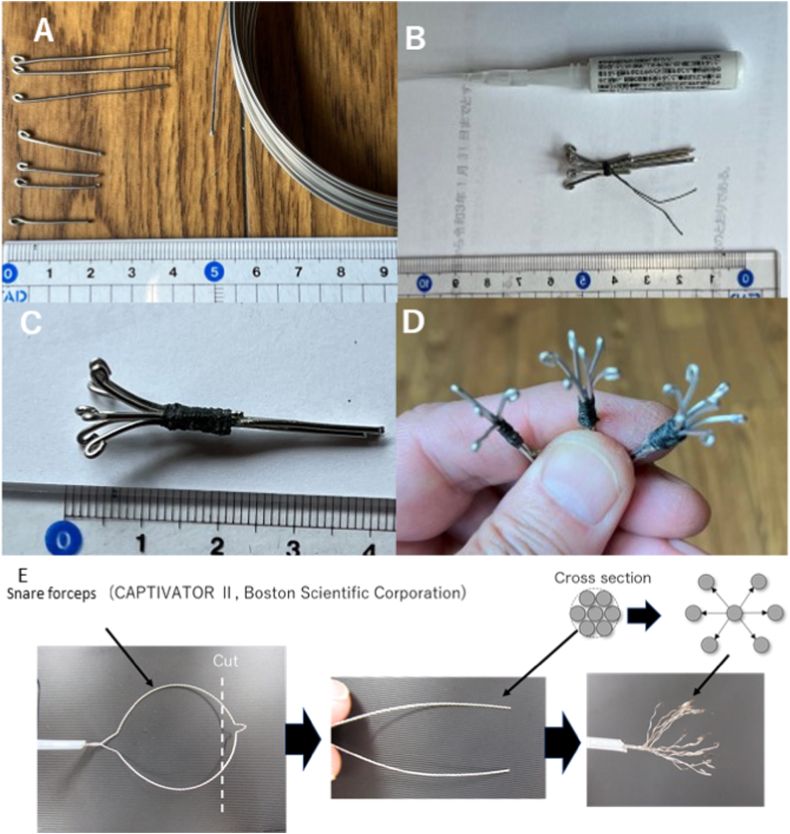
Creation of a multi-spray tip A: The steel wire used to create the tips is shown. B: The tips are temporarily fixed using silk thread and reinforced using Aron Alpha. C: A completed Spray 7 tip is shown. D: Forceps tips with four, five, and seven spray tips are shown. E: The tip of the snare-forceps (CAPTIVATOR II 15 mm, Boston Scientific Corporation) is modified to create a pair of forceps that can be manually opened and closed using the endoscope tip.

Stainless steel wire tips (diameter: 1 mm) were rounded using radio pliers and cut in lengths of 2 and 4 cm (Fig. 1A). Three 4-cm wires were bundled in the central region, the 2-cm wire tips were aligned with silk thread, and the surrounding areas were fixed with superglue (Fig. 1B). A single wire was arranged in the center and six wires were arranged around the periphery to form a circular tip with a long diameter of 8 mm (Fig. 1C). The seven-wire tip was termed ‘Spray 7.’ Six- and four-tip spray ends were created to determine the effect of the number of spray tips (Fig. 1D).

In addition, the tip of the snare forceps device (CAPTIVATOR II 15 mm, Boston Scientific Corporation, Marlborough, MA, USA) was modified to create a pair of forceps that can be opened and closed manually using the endoscope tip (Fig. 1E).

The multi-tip spray tips were designed for insertion into the tip of a reusable, manually controlled scalpel holder. A Valleylab Force FX (Valleylab Inc., Boulder, CO, USA) was used as a high-frequency generator.

### 2.2 Mini-pig experiment

One adult male micro-mini-pig (MMP; Fuji Micra, Shizuoka, Japan; body weight: 23.7 kg; age: 37 months) was used. The MMP was maintained on a diet of infant milk and permitted to drink water ad libitum and was fasted for 24 h preoperatively. The experiment was approved by the Kitasato University (approval number: 21–087) and was conducted in accordance with the experimental animal guidelines and experimental animal breeding management manual.

The MMP was intubated under full anesthesia. The liver was exposed via an inline incision in the upper abdomen, and the lateral segment was transected using a scalpel. Hemostasis was attempted using a conventional one-tip claw (monopolar cautery) and Spray 7. The spraying was conducted by an expert with over 30 years of experience in liver surgery.

The MMP was sacrificed via exsanguination in the thoracic cavity using sufficient anesthesia. The liver, kidneys, and stomach were immediately harvested for ex vivo experiments. Other pigs were also sacrificed, and partial kidney resections were performed with a scalpel during laparotomy. Hemostasis was attempted using variable spray biopsy forceps through a laparoscope.

### 2.3 Ex vivo experiments

A saline-moistened gauze was placed on a metal return electrode, and the organ to be tested was placed atop the gauze. A spray experiment was conducted by connecting the high frequency-generating power supply and changing the tip of the monopolar cautery device. The excised organ was moisturized using warm physiological saline.

The spray range of surgeons with different levels of experience was compared between an expert with 40 years of surgical experience in human clinical fields (EK), an operator with 25 years of veterinary clinical experience (SI), and a person with no experience (SK). The spraying of the excised liver surface (lasting approximately 2 s at 30 W or 60 W) was conducted five times using a conventional monopolar cautery spray end and Spray 7. The cauterization areas were compared using photographs.

The effect of the number of tip spray ends on the exposure area and depth was also investigated. The excised stomach was opened, and the mucosal surface was sprayed at 30 W and 60 W for 2 s using a conventional one-tip claw (monopolar cautery), four tips, and seven tips. The cauterized specimen was fixed in formalin and investigated pathologically.

Spray 7 was subsequently used to expose the kidney surface to different types of electric power from the medulla side. The time required for tissue cauterization and necrosis was measured, and the extent of cauterization from the surface was pathologically investigated.

The speed and coagulation ability of wide-area cauterization using Spray 7 was determined by immobilizing the operator (SK), creating a liver transection with a scalpel, and measuring the macroscopic cauterization time with one terminal (30 W) on one side and Spray 7 (60 W) on the other side. This was repeated four times, and the time required for total cauterization was measured, and the cauterization surface was analyzed pathologically.

### 2.4 Histopathological evaluation

The hematoxylin and eosin staining was conducted by Sept Sapie Co. Ltd. (Tokyo, Japan). An experimental pathology specialist interpreted the results using the following evaluation method.

## 3 Evaluation method

### 3.1 Stomach

#### 3.1.1 Height of the normal mucosa adjacent to the cauterization damage site (μm)

The distance from the mucosal surface to the muscularis mucosae was defined as the height of the normal mucosa adjacent to the cauterization site. The height was measured at four arbitrary locations and the average value was used in the analyses.

#### 3.1.2 Height of the non-invasive mucosa at the cauterization site (μm)

The boundary of the cauterized, non-invasive tissue was defined as the deepest irreversible denaturation (gastric corpus: condensation of main cells; pyloric part: condensation of gastric pit epithelial cells), and the distance from the deepest point of that boundary to the muscularis mucosae was defined as the height of the non-invasive mucosa of the cauterization site. The height was measured at four arbitrary locations and the average value was used in the analyses.

### 3.2 Cauterization damage (invasion; μm)

The cauterization damage was determined by subtracting the height of the non-invasive mucosa at the cauterization site from the height of the normal mucosa adjacent to the cauterization damage site.

### 3.3 Kidney

The deepest part of the cauterization damage (invasion) was defined as the deepest part of the irreversible denaturation (condensation of tubular epithelial cells). The length from the capsule to its deepest part was defined as the depth of the cauterization damage (invasion). This was measured at four arbitrary locations and the mean and standard deviation were used in the analyses.

### 3.4 Liver

Areas with abnormal findings of hepatocyte nucleus swelling and hepatocyte condensation were targeted. The length from the superficial surface of the target lesion to the deepest part was used as an index of the depth of cauterization damage (invasion). This was measured at eight arbitrary locations and the mean and standard deviation were used in the analyses.

### 3.5 Statistical analysis

The Mann-Whitney *U* test was conducted and 95% confidence intervals were determined using the R statistical analysis software (https://cran.r-project.org/, The Institute of Statistical Mathematics, Tachikawa, Tokyo, Japan).

## 4 Results

### 4.1 In vivo experiment

The scalpel transection of the liver is shown in Video A. The conventional monopolar cautery spray cauterization (Video B) was followed by the use of Spray 7 (Video C). No hemostasis was achieved after approximately 40 s of cauterization using the monopolar cautery spray. Hemostasis was achieved in approximately 35 s when Spray 7 was used.

### 4.2 Ex vivo experiment

#### 4.2.1 Evaluation of differences in spray area due to differences in surgical experience

**Fig. 2:**
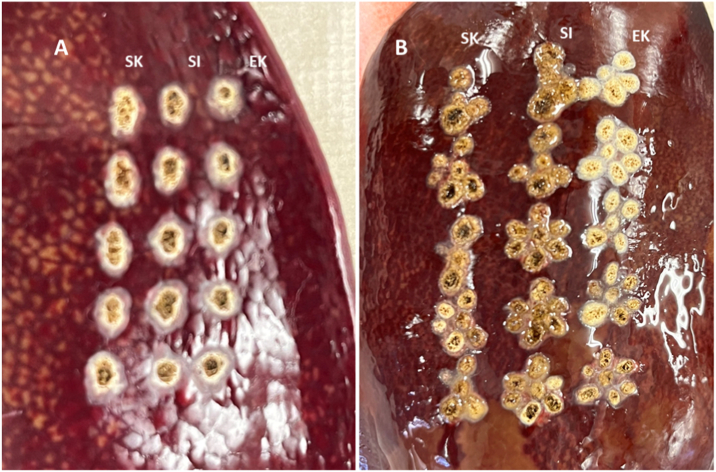
Spray coagulation of the surface of an explanted liver A: Single-tip spray B: Seven-tip spray.

The cauterization area was similar with both the conventional, one-tip, 30-W spray and the 60-W spray used by experts. However, the individual techniques differed between expert and inexperienced users (Fig. 2A). When Spray 7 was used at 30 W, the differences were more significant; however, no inter-individual variability or variability due to differences in proficiency was observed at 60 W (Fig. 2B).

#### 4.2.2 Impact on gastric mucosal surface

**Fig. 3:**
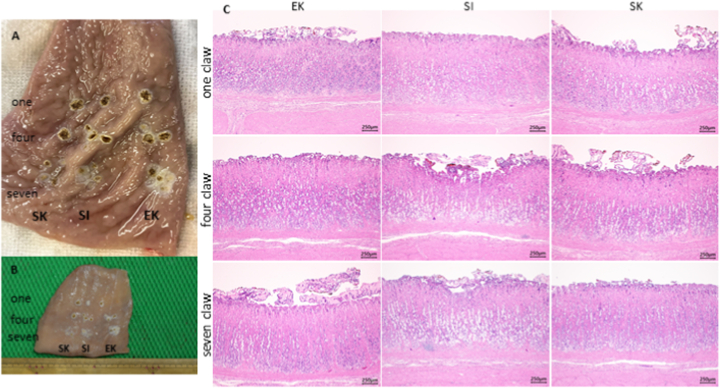
Cauterization of the mucosal surface of a stomach A: Raw specimen; macroscopic findings immediately after cauterization of gastric mucosa (60 W) (From top: one claw, four claws, seven claws, from left: EK, SI, and SK) B: Fixed specimen; macroscopic findings immediately after cauterization of gastric mucosa (60 W) (From top: one claw, four claws, seven claws, from left: EK, SI, and SK) The cauterization depth was deep for all operators with a one-tip claw; however, the cauterization site was broad and uniform with a seven-tip claw. C: Histopathological specimen (HE) after cauterization of gastric mucosa (60 W) There was a tendency for the depth of cauterization damage (invasion) to be deeper with the high-output spray (60 W) than with the normal-output spray (30 W), regardless of operator. With normal contraction force and high output, experienced operators could cauterize more uniformly and with less damage.

The cauterization depth of all operators using a one-tip claw at 60 W was similar; however, the cauterization site was broad and uniform when Spray 7 was used. The disappearance of the gastric mucosal epithelial cells was increased when more terminals were used, allowing for the cauterization of the mucous membrane over a wide range after a single cauterization (Fig. 3A–C).

The depth of cauterization damage (invasion) tended to be deeper with the high-output spray (60 W) compared to the normal-output spray (30 W), regardless of the operator (Table 1A and B). The expert operator (EK) achieved uniform cauterization with less damage when Spray 7 was used at 60 W (Table 1B).

**Table 1A:**
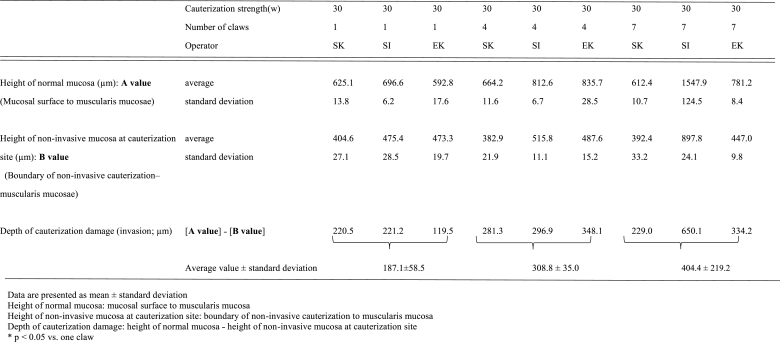
Histological measurement data of mini-pig gastric mucosa (pyloric area).

**Table 1B:**
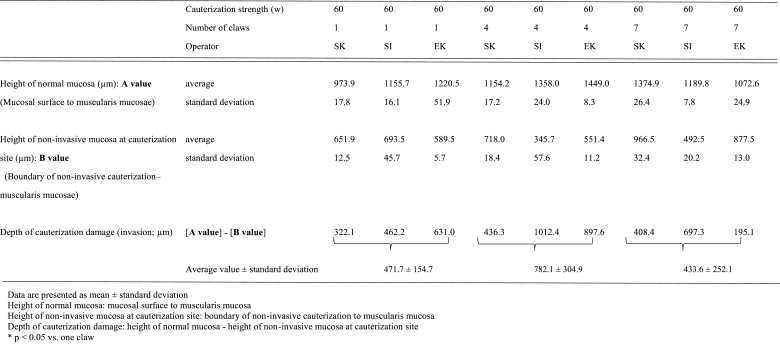
Histological measurement data of mini-pig gastric mucosa (gastric corpus).

The depth of cauterization damage (invasion) was not significantly different when the one-tip claw and Spray 7 devices were used at 60 W (p < 0.05; Table 1B).

An overall loss of mucosal epithelial cells (exfoliation) was observed at the cauterization site, and denaturation (cell concentration, nuclear condensation, and cytoplasm homogenization) was observed in the adjacent cells that were deeper than the cauterization site (Table 2A, Table 2B).

**Table 2A:**
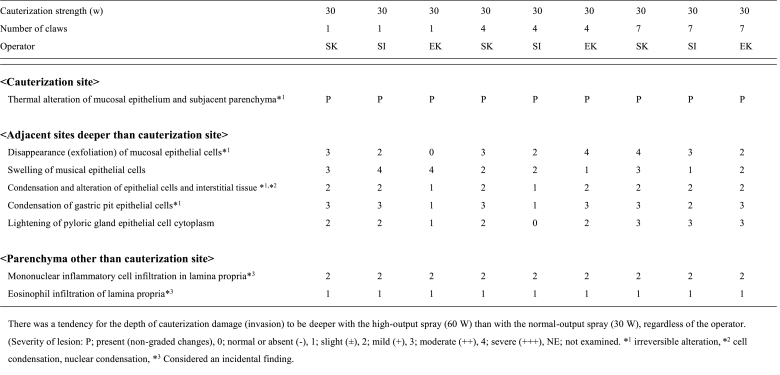
Histological findings of mini-pig gastric mucosa (pyloric area).

**Table 2B:**
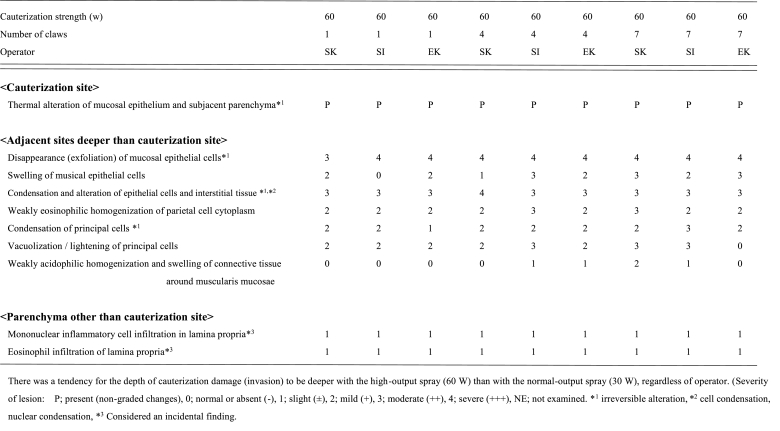
Histological findings of mini-pig gastric mucosae (gastric corpus).

#### 4.2.3 Influence of electric power level on kidney tissue coagulation and necrosis

**Fig. 4:**
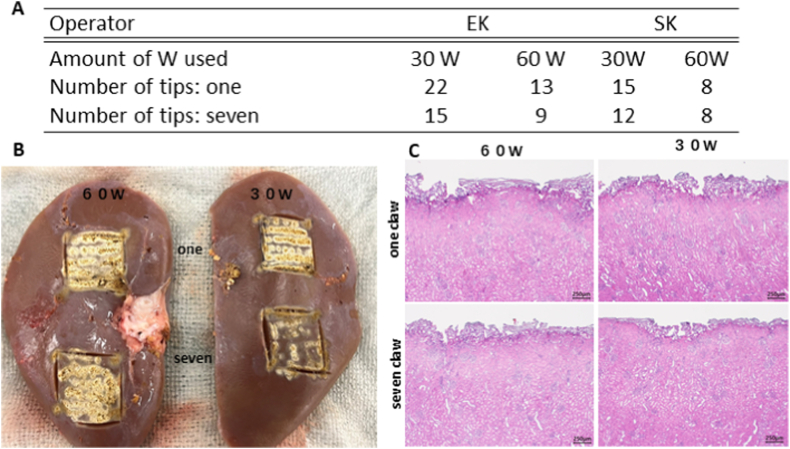
Cauterization of the capsular surface of kidneys A: The cauterization time of a limited area Increased electric power resulted in a shorter cauterization time, regardless of the experience of the operator. The cauterization time for experienced operators decreased as the number of spray tips increased. B: Macroscopic findings after cauterization A single tip achieved a more uniform cauterization. C: Histopathological specimen after cauterization The denaturation of the renal tubular epithelium and Bowman's capsule was observed after cauterization of the renal capsule.

Increased electric power resulted in a shorter cauterization time, regardless of the experience of the operator (Fig. 4A). Additionally, the cauterization time of experienced operators decreased as the number of spray tips increased.

A single tip allowed for a uniform cauterization of the kidney; however, the cauterization time was longer than that of X (Fig. 4B).

The depth of cauterization achieved using the one-claw device was significantly deeper at 60 W than that at 30 W (668.8 ± 110.5 vs. 993.5 ± 37.7; p = 0.002857; Table 3A). In contrast, there was no significant difference in the depth of ablation when Spray 7 was used at 60 and 30 W (551.4 ± 48.4 vs. 576.9 ± 48.9). When Spray 7 was used at 60 W, the tissue damage was reduced, and the ablation time was shortened (Table 3A and Fig. 4B).

**Table 3A:** Histological measurement data of mini-pig kidney.

Cauterization strength(w)	30	60	30	60
Number of claws	1	1	7	7
**from estimated capsule line to finding (μm)**				
Average value ± standard deviation	668.8 ± 110.5	993.5 ± 37.7^#^	551.4 ± 48.4	576.9 ± 48.9

#v.s. one-claw at 30 W p < 0.05 using.

The denaturation of the renal tubular epithelium and Bowman's capsule was observed after cauterization (Table 3B and Fig. 4C).

**Table 3B:** Histological measurement data and histological findings of mini-pig kidney.

Cauterization strength(w)	30	60	30	60
Number of claws	1	1	7	7
**<cauterization site>**				
Thermal alteration of capsule and parenchyma just below capsule*^1^	P	P	P	P
**<Adjacent sites deeper than cauterization site>**				
Condensation of renal tubular epithelial cells*^1,^*^2^	2	3	1	2
Slight swelling/alteration of Bowman's capsule wall	1	1	1	1
Eosinophilic homogenization of tubular epithelial cell cytoplasm	2	3	2	2

Severity of lesion: P; present (non-graded changes), 0; normal or absent (−), 1; slight (±), 2; mild (+), 3; moderate (++), 4; severe (+++), NE; not examined. *^1^ irreversible alteration, *^2^ cell condensation, nuclear condensation.

#### 4.2.4 Cauterization/coagulation time and pathology of the transection surface of the excised liver

**Fig. 5:**
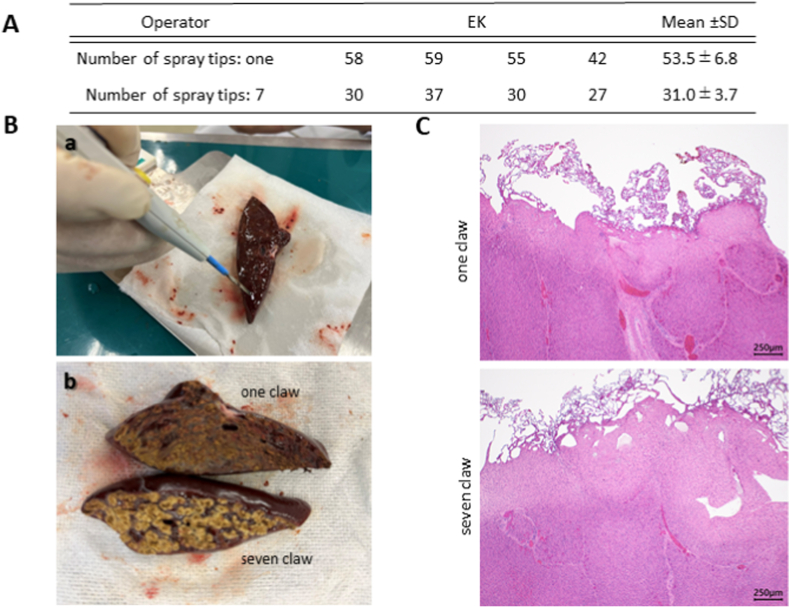
Cauterization of the excised liver tissue A: The spray cauterization time of the liver transection surface from operator fixation Spray 7 reduced the cauterization time by half. B: Cauterization of the liver surface a. Appearance of cauterization of liver surface The contact area is small, and the cauterization time is long when a one-claw device is used. b. Macroscopic findings after cauterization of the liver surface The conventional single-terminal spray achieved uniformity, whereas Spray 7 required less time. C: Histopathological specimen after cauterization Pathological examinations revealed thermal denaturation of the surface tissues, swelling of hepatocyte nuclei, condensation and degeneration of hepatocytes, and condensation of the bile duct epithelium after cauterization at 60 W.

The use of Spray 7 required approximately half the amount of time than that with the use of X device (Fig. 5A). In macroscopic findings after cauterization of the liver surface, the conventional single-terminal spray achieved cauterization in a relatively uniform manner, whereas Spray 7 required less time (Fig. 5B–a, b).

The mean cauterization depths were 371.8 ± 75.5 μm and 218.3 ± 89.8 μm when the one-claw and Spray 7 devices were used, respectively (p = 0.002953) (Table 4)

**Table 4:**
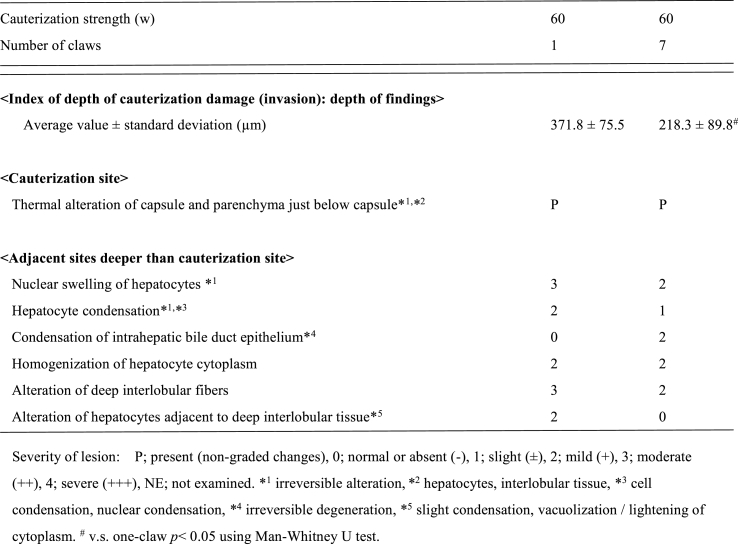
Histological measurement data and histological findings of mini-pig liver.

Thermal denaturation damage of the surface tissues, swelling of hepatocyte nuclei, condensation and degeneration of hepatocytes, and condensation of the bile duct epithelium were observed (Fig. 5C).

#### 4.2.5 Practice of variable multivessel spraying through the hole of endoscopic biopsy forceps

Under laparoscopic conditions, the lower portion of the left kidney was partially resected using a scalpel. Spray hemostasis was performed using a homemade variable multi-tip spray forceps through the forceps hole while holding the endoscope, and primary hemostasis was achieved in the same area using gauze (Video D). The tip was flexible, and hemostasis was achieved via cauterization at 40 W.

## 5 Discussion

This study presents a novel spray tip that enhances the function of a high-frequency power supply by altering the tip of a monopolar electric scalpel from a single tip to one with seven spray electrode terminals.

Spray 7 achieved hemostasis in a shorter time compared with the conventional spray device in an in vivo transected MMP liver. The use of a snare spray to achieve hemostasis has been investigated in porcine models with gastric antrum telangiectasia [6]. Hemostasis can be achieved over a wider area when multiple tips are used than with a single-tip spray.

The use of the conventional single- and multiple-tip sprays by three operators with various levels of experience was investigated using organs harvested from a mini-pig. When the number of spray tips increased, the cauterization effect improved as the electric power increased. This improvement in the cauterization effect may reduce the variability due to operator experience.

The coagulation and cauterization depths were previously investigated in the mucosal surface of a resected pig stomach [5]. In this study, the use of Spray 7 resulted in a more uniform cauterization and coagulation depth than that with the use of a normal one- or four-tip spray device. When the four-tip spray device was used, the deviation in the distance to the mucous membrane due to the inclination of the spray tip might have led to the differences in the transmission of the cauterization power.

Furthermore, pathological examinations of the cauterization of parenchymatous organs indicated that, when spraying from the cortex of the kidney, cauterization could be conducted rapidly and without variation even when the spray power capacity of Spray 7 was twice that of the one-tip device. A uniform coagulation was achieved without variation of the cauterization depth even when the power was increased to 60 W.

In the non-contact coagulation, the electric power is concentrated at one point in a single tip, resulting in high heat and evaporation of proteins. This causes desiccation, vaporization, or charring of the target tissue, which spreads to deeper tissues [7]. The monopolar tips are capable of hemostasis in vessels ≤2 mm in diameter; however, its impact on surrounding tissues can reach up to 2 cm [8,9]. Spray 7 has a nearly circular shape, which increases the uniformity of the contact area and reduces the damage to deeper tissues, thus resulting in the hemostatic effect with a shorter time to hemostasis.

Although electrical devices have several advantages, they may result in adverse events [[10], [11], [12]]. Burns to structures other than the surgical site, such as the return electrode, implants, or gastrointestinal tract, and burns to operators or nurses, are major problems encountered not only in general surgery but also in endoscopic surgery [[13], [14], [15]]. These can be prevented with the knowledge of appropriate applicability of electrical devices, new technologies, and potential dangers [16]. However, surgeons may not be familiar with these devices, despite using them frequently [17,18]. The novel tip described in this study allows for a uniform cauterization by operators of all skill levels, reduces damage to deeper tissue, shortens the hemostasis time, and may reduce the occurrence of complications. This tip can be used with new devices and other electrical devices used for simulations to limit the occurrence of complications and for training purposes [2].

The multiple-tip spray device presented in this study can be used to achieve a simple and safe hemostasis. Laparoscopic hemostasis at the cut stumps of the parenchymatous organs is important and has been investigated in porcine models [19]. In this study, the safety and efficacy of this novel multi-tip spray device was demonstrated. The opening area of the device can be altered via the installation of a spring action in the tip (Fig. 1). In addition, hemostasis could be achieved using a soft laparoscope as forceps. This technique can be used in specular and robotic surgeries. The use of this new device is expected to provide significant benefits to practitioners and patients during surgeries involving parenchymal organs, including the liver and kidneys. The use of this device may allow for shorter operative times, less blood loss, less pain, and less invasive surgeries as it allows for a shorter hemostasis time and lesser tissue damage than the conventional monopolar cauterization. In addition, the patients’ postoperative quality of life may be improved.

This study also had some limitations. The incidences of complications associated with Spray 7 and those associated with general electric scalpels were not different. However, the position and area of the electrode plate must be confirmed in vivo to avoid complications of burns when monopolar electrodes are used. The occurrence of complications, such as burns at the location of the return electrode due to a high-frequency power supply, was not assessed in this study and requires further investigation. In addition, this device may likely cost more than the conventional monopolar devices; however, the cost was not investigated in this study. Furthermore, although studying the effect on hemostasis using a live pig was an advantage, the effect on the wound immediately after awakening from anesthesia and during the postoperative period for several days after surgery was not investigated. Therefore, it may be necessary to observe the effect during the perioperative period in the future.

## Funding

This work was supported by the Kobayashi Regenerative Research Institute, LLC. [Recipient: Eiji Kobayashi, Grant number: none].

## Data availability statement

All relevant data supporting the findings of this study are either included within the article or are available upon request from the corresponding author.

## Contribution statement

Conceived and designed the experiments: Satomi Iwai, Shou Kobayashi, Eiji Kobayashi.

Performed the experiments: Satomi Iwai, Shou Kobayashi, Shinji Torai, Eiji Kobayashi.

Analyzed and interpreted the data: Satomi Iwai, Shinji Torai, Eiji Kobayashi

Contributed reagents, materials, analysis tools or data: Satomi Iwai, Shou Kobayashi, Eiji Kobayashi.

Wrote the paper: Satomi Iwai, Shou Kobayashi, Shinji Torai, Eiji Kobayashi.

## Declaration of competing interest

The authors declare the following financial interests/personal relationships which may be considered as potential competing interests:

Research Support:

Eiji Kobayashi reports that financial support was provided by Kobayashi Regenerative Research Institute, LLC. Wakayama, Japan.

The funder had no role in the study design, data collection and analysis, decision to publish, or preparation of manuscript.

Relationships:

1. Eiji Kobayashi reports a relationship with SCREEN Holdings Co., Ltd. Kyoto, Japan that includes: consulting or advisory.

2. Eiji Kobayashi reports a relationship with Kobayashi Regenerative Research Institute, LLC., Wakayama, Japan that includes: board membership.

3. Shinji Torai reports a relationship with SCREEN Holdings Co., Ltd. Kyoto, Japan that includes: employment.

4. Shou Kobayashi reports a relationship with Kobayashi Regenerative Research Institute, LLC. Wakayama, Japan, that includes: employment.

Patents and Intellectual property:

1. Satomi Iwai has patent pending to SCREEN Holdings Co., Ltd.

2. Shou Kobayashi has patent pending to SCREEN Holdings Co., Ltd.

3. Shinji Torai has patent pending to SCREEN Holdings Co., Ltd.

4. Eiji Kobayashi has patent pending to SCREEN Holdings Co., Ltd.

Other activities:

There are no other relationships or activities to declare.
